# Investigation of the Inhibitory Effects of Mangrove Leaves and Analysis of Their Active Components on Phaeocystis globosa during Different Stages of Leaf Age

**DOI:** 10.3390/ijerph15112434

**Published:** 2018-11-01

**Authors:** Min Zhao, Han Xiao, Dong Sun, Shunshan Duan

**Affiliations:** Department of Ecology, College of Life Science and Technology, Jinan University, Guangzhou 510632, China; 13250727965@163.com (M.Z.); acehsiaohan@gmail.com (H.X.); jnu_sundong@163.com (D.S.)

**Keywords:** water pollution, HABs, mangrove plants, inhibitory effects, chemical composition

## Abstract

The presence of harmful algal blooms (HABs) can cause significant problems to the quality of the water, the marine ecosystems, and the human health, and economy worldwide. Biological remediation can inhibit harmful algal growth efficiently in an environmental-friendly manner. Therefore, the research conducted on biological remediation with regard to the inhibition of HABs is becoming a major focus in marine ecology. To date, no study has been reported with regard to the red tides occurring in mangrove wetlands. Therefore, the present study used two mangrove species, namely *Bruguiera gymnorrhiza* and *Kandelia candel* and one harmful algae species *Phaeocystis globosa* as experimental organisms. The present study determined the inhibitory effects and algae physiology of specific aqueous extracts from mangrove leaves on the viability of harmful algae, and analyzed the main chemical composition of the aqueous extracts by ultra-performance liquid chromatography coupled to high resolution mass spectrometry (UPLC-QTOF-MS). The results indicated that the aqueous extracts from different leaf ages of *B. gymnorrhiza* and *K. candel* leaves exhibited apparent inhibitory effects on the growth of *P. globosa*. The inhibitory effects of *B. gymnorrhiza* and *K. candel* leaves aqueous extracts on the growth of *P. globosa* were in the following order: senescent > mature > young leaves. The levels of the parameters superoxide dismutase (SOD) activity, glutathione (GSH), and malondialdehyde (MDA)content in *P. globosa* following treatment with *B. gymnorrhiza* and *K. candel* leaves aqueous extracts were increased as follows: senescent > mature > young leaves. Simultaneously, the intensity of the ion peaks of the specific secondary metabolites assigned **4** (No.: 4 Rt: 2.83 min), **7** (No.: 7 Rt: 3.14 min), **8** (No.: 8 Rt: 3.24 min), **9** (No.: 9 Rt: 3.82min) and **10** (No.: 10 Rt: 4.10 min) were increased. These metabolites were found in the aqueous extracts from *B. gymnorrhiza* leaves. The intensities of the ion peaks of the secondary metabolites **7**, **8** in the aqueous extracts from the *K. candel* leaves were also increased. The majority of the substances that inhibited the algae found in the mangrove plants were secondary metabolites. Therefore, we considered that the norsesquiterpenes compounds **4**, **8**, **9**, and **10** and a phenolic glycoside compound **7** were the active constituents in the aqueous extracts of the mangrove leaves responsible for the inhibition of algae growth. This evidence provided theoretical guidance for the development of biological methods to control red tides and for the further use of substances with antiproliferative activity against algae.

## 1. Introduction

Harmful algal blooms (HABs), also known as red tide, are mainly caused by water eutrophication and are considered an increasingly severe environmental problem in oceans [1]. The prevention of this environmental problem requires significant funds, whereas the water quality, fishery resources and human health can be damaged by red tides [2,3]. Harmful algal blooms can reduce the quality of water bodies and thus hinder the use of water resources in several different ways [4]. For example, the outbreak of *Phaeocystis globosa* (*P. globosa*) can form foams on the sea surface, seriously affecting the water quality [5]. In addition, the ecological environment and economic development are severely affected [5]. During 2014–2016, the detection of harmful algal *Pseudo nitzschia* occurred on the west coast of North America, with extensive ecological and economic impacts [6]. Red tide algae can further produce shellfish poisoning, due to shellfish ingestion of the algae. The toxins are enriched in the shellfish and this can result in poisoning from shellfish consumption [7]. The series of these problems that are caused by HABs have attracted the interest of the scientific community in order to develop possible means of preventing these outcomes.

The methods for the inhibition of harmful algae growth include physical [8], chemical [9], and biological remediation [10]. Physical and chemical remediation are not widely used due to water pollution and high cost [11,12]. Moreover, biological remediation mainly utilizes nutrient competition, ingestion, and inhibition by other organisms in order to reduce the biological density of the red tide algae and/or to inhibit algal cell viability [13]. The use of biological remediation to inhibit algal growth, such as vegetation, is efficient and environmentally friendly. Therefore it has become an increasingly popular research area [14]. Various studies have been conducted on algae inhibition and these have examined the use of terrestrial plants [15], aquatic plants [16], and algae [17]. Due to the several advantages of biological remediation, considerable attention has been paid to its use for the inhibition of harmful algae.

Mangroves are considered parts of a woody plant community in tropical and subtropical coastal intertidal zones with high ecological value [18]. Several mangrove plants produce chemicals with inhibitory effects on other plants and such inhibitory effects have been widely reported [19,20]. Moreover, various mangrove plant leaves have shown inhibitory effects on red tide algae [21]. Certain studies have shown that both dry powder aqueous extracts and fresh tissue aqueous extracts of mangrove leaves can significantly inhibit microalgae growth [22]. The dry powder aqueous extracts of the leaves of the mangroves *Rhizophora apiculata*, *Bruguiera sexangula*, *Ceriops tagal*, *Acanthus ilicifolius*, *Acrostichum aureum*, and *Cerbera manghas* have shown significant inhibitory effects on the growth of *Skeletonema costatum* [23]. To date, no study has been reported with the red tides occurring in mangrove wetlands.

The present study used aqueous extracts as the basic condition to analyze the chemical constituents of the mangrove leaves, which were different from other research methods that used organic solvents to directly soak the plants and extract chemical components [24]. For example, phenolic compounds can be extracted with acetone from Guayusa leaves [25]. A total of three iridoids and seven polyphends were identified in sesame leaves by ethanol extraction [26]. Quinic acid was identified in Marula leaves by ethanol extraction [27]. The use of organic solvents to extract chemical constituents in plants can increase the extract content of the chemical components, however the substances that can be extracted may not be identical to the substances that plants release into the surrounding environment and responsible for inhibition on algae under natural conditions. Under natural conditions, living plants release chemicals into the environment by root exudation [28], stem and leaf leaching, and volatilization [29]. Therefore, the chemical components extracted by water can more accurately reflect the chemical composition that inhibits the growth of algae cells in the plants.

During exposure of algal cells to an inhibitory effect, the oxygen is converted into a large amount of active oxygen [30]. The active oxygen and its products have strong oxidizing capacity and a degrading effect on several biomolecules [31]. Superoxide dismutase (SOD) is the enzyme, which belongs in the antioxidant enzymatic system and can scavenge reactive oxygen in algal cells. Furthermore, glutathione (GSH) is an important factor used in the measurement of the antioxidant capacity of the algal cells, whereas the content of malondialdehyde (MDA) in algal cells can identify the severity of attack by active oxygen [32]. A series of studies have used these indicators to reflect the physiological state of algae [33,34,35].

*Phaeocystis globosa* is a representative species of harmful algae that can cause large-scale HABs in several regions worldwide [5]. In the present study, *P. globosa* was selected as a representative species of red tide algae, whereas two mangrove species of *Kandelia candel* and *Bruguiera gymnorrhiza* were selected to investigate the effects of different leaf ages on the growth and antioxidant capacity of *P. globosa* under laboratory conditions. In order to further investigate the inhibitory mechanism of *K. candel* and *B. gymnorrhiza* leaves on harmful algae, ultra-performance liquid chromatography coupled with high resolution mass spectrometry (UPLC-QTOF-MS) was used to identify the chemical composition of the aqueous extract from the mangrove leaves. Furthermore, the chemical constituents of the mangrove leaves during different leaf ages were compared in order to examine the inhibition of algae growth and to determine the mechanism involved. The present study provided theoretical guidance for the development of biological methods to control red tides, improve red tide-induced water pollution, and identify growth inhibitory substances for algae.

## 2. Materials and Methods

### 2.1. Phytoplankton Cultures

Prior to the experiment, experimental alga *P. globosa* was obtained from the Research Center of Hydrobiology, Jinan University, Guangzhou, China, and grown in 200-mL Erlenmeyer flasks containing sterilized artificial seawater with f/2 medium [36,37]. The algal cells were cultured under conditions of 4000 lux, 23 ± 1 °C, 12-h light/dark cycle. The cell flasks were shaken on the shaker (Shaker speed: 80 rpm) three times a day for 10 min each time. The suspensions were inoculated into new Erlenmeyer flasks at the log phase, and this step was repeated four times for algal cell activation.

### 2.2. Leaf Collections

**Figure 1:**
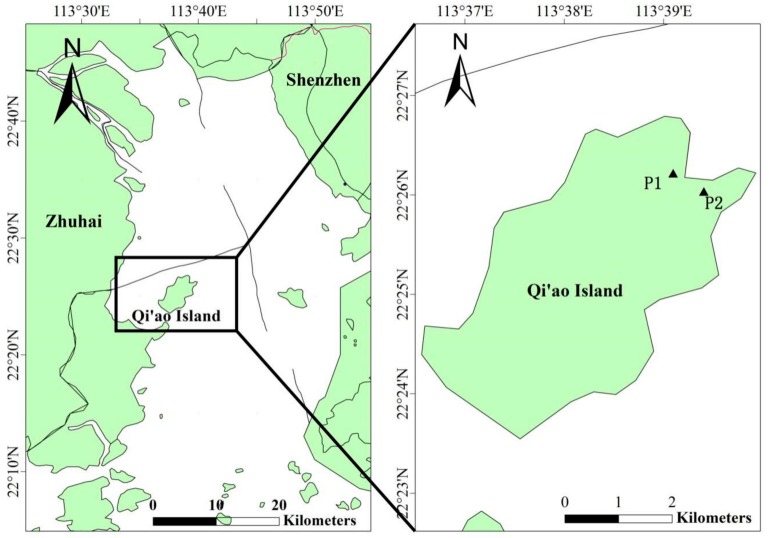
Map of the sampling sites. (P1: Community of *K. candel*; P2: Community of *B. gymnorrhiza*).

Since mangroves are tropical and subtropical evergreen broad-leaved plants, the mangrove leaves keep falling in the whole year. Prior to the start of the experiment, we observed the growth cycle of *K. candel* and *B. gymnorrhiza* leaves and found that the leaves were grown out from the top of the branches. The leaves were continuously grown until reaching the trunks, indicating that the leaves at the top of the branches were newly grown leaves. Therefore, leaf samples of mangrove plants were collected according to the study by Lin et al. [38] with some modifications. The leaves were collected from the Qi’ao Island (22°23′34″–22°26′38″ N, 113°36′40″–113°40′33″ E) in the Zhuhai, Guangdong on the 20 June 2017 (Figure 1). In the mangrove community, 500 g of young, mature, and senescent leaves were separately collected from the mangrove plants of the same age, height, and growth. During sampling, branches with 6 and 8 pairs of leaves were selected for *K. candel* and for *B. gymnorrhiza*, respectively. The leaves that were degraded by insects or mechanically damaged were avoided. The young leaves comprised the first pair of small green leaves from the branch top (grow out 1–5 days). The collected *B. gymnorrhiza* leaves were 11 ± 1 cm in length and 4.5 ± 0.5 cm in width, whereas the *K. candel* leaves were 5.5 ± 0.5 in length and 2.5 ± 0.5 cm in width. Mature leaves were the fourth pair of leaves from the top (growth within 15–30 days), and were dark green and fully stretched out. The *B. gymnorrhiza* leaves that were collected were 16.5 ± 0.5 cm in length and 7.5 ± 0.5 cm in width, whereas the *K. candel* leaves were 14.5 ± 0.5 in length and 6.5 ± 0.5 cm in width. Senescent leaves were yellow and were growing to the nearest regions of the trunks (growing time for more than 45 days).

### 2.3. Preparation of Aqueous Extracts from Mangrove Leaves

The leaf preparation method was conducted according to Sun et al. [21] with some improvements. The fresh leaves were washed with distilled water and put the leaves into drying oven, then air-dried at temperature 25 °C for 1 week to a constant weight. The leaves were crushed with a plant mill and filtered with a sieve in order to collect dried leaf powder. A total of 10 g of dry powder was accurately weighed and placed in a sterile 200-mL Erlenmeyer flask with 100 mL of sterilized artificial seawater. The bottle mouth was sealed with laboratory film. The bottles were kept at room temperature for 48 h, and gently shaken three times a day. Subsequently, the aqueous extracts were centrifuged (5000 rpm for 15 min) and the supernatant filtered with a disposable syringe filter to remove unwanted microorganisms. The concentration of the aqueous extracts was 100 g·L**^−^**^1^.

### 2.4. Experimental Setup

2.5 mL of different leaf age of *B. gymnorrhiza* leaf dry powder aqueous extracts (100 g·L**^−^**^1^) were respectively added to 47.5 mL of algae culture in a 100-mL Erlenmeyer flask. This resulted to a final concentration of 5 g·L**^−^**^1^ of the extract. A total of 2.5 mL of sterilized artificial seawater was added to 47.5 mL of algae culture as a control and 4 mL of different leaf age of *K. candel* leaf dry powder aqueous extracts (100 g·L**^−^**^1^) were respectively added to 46 mL of algae culture leading to a concentration of 8 g·L**^−^**^1^ of the extract of *K. candel* leaves. A total of 4 mL of sterilized seawater was added to 46 mL of algae culture as a control. The initial inoculation density of the algae culture was 1.97 × 10^5^ cells·mL**^−^**^1^. All the experiments above were conducted in triplicate. All flasks were placed in an artificial climate box.

### 2.5. Bioassays

A total of 40 mL of algae culture was placed in a 50-mL centrifuge tube and centrifuged at 3500 rpm for 10 min. The supernatant was discarded. The collected algal cells were weighed and according to the weight (g): volume (mL) ratio (1:9), phosphate buffer (10 mM, pH 7.4) was added to the collected algal cells. The algal cells were disrupted in an ultrasonic cell disrupter to a final 10% suspension. Following centrifugation at 4 °C and 3000 rpm, the culture supernatant was collected and stored in a refrigerator at 4 °C until further use.

The MDA and GSH contents and the SOD activities were measured using commercial kits (Nanjing Jiancheng Bioengineering Institute, Nanjing, Jiangsu, China). The MDA content was measured based on the method of Ohkawa et al. [39]. The principle of the MDA content determination was based on the conversion of MDA to thiobarbituric acid in order to form a red product with a maximum absorption peak at 532 nm. The homogenate supernatants were mixed with reagents, placed in a boiling water bath for 1 h and finally centrifuged. The absorbance values were measured at 532 nm. The MDA content in the algal cells was expressed as nmol·mg**^−^**^1^ protein (prot). A standard curve of the MDA content was constructed and used to determine the sample MDA content.

The activity of SOD was determined based on the method of Du et al. [40]. The principle of the assay was based on the production of superoxide radical by the xanthine oxidase reaction system. This oxidizes hydroxylamine to form nitrate, which in turn is converted to a reddish purple color under the action of a chromogenic reagent. A total of 200 μL of the homogenate supernatant of the SOD standard was added to a 96-well plate with 20 μL of the sample and incubated at 37 °C, for 20 min. The absorbance was measured at 450 nm. The SOD activity in algal cells was expressed as U·mg**^−^**^1^ prot.

The GSH content was measured based on the method of Zhang and Meng [41]. The principle of the assay was based on the reaction of GSH with dithionitrobenzoic acid that produces a yellow compound, which can be measured at 405 nm. Following mixing of the sample homogenate with the reagents, the samples were kept for 5 min at room temperature, and subsequently the GSH content was determined by measuring the absorbance at 405 nm. The GSH content was expressed as µmol·g**^−^**^1^ protein. All the experiments were repeated three times.

### 2.6. Chemical Composition Analysis of Aqueous Extract from Mangrove Leaves

#### 2.6.1. Chemical Composition Analysis

Ultra Performance Liquid Chromatography (UPLC) analysis was carried out on an Acquity UPLC I-class system (Waters Corporation, Milford, MA, USA), with an Acquity BEH C18 (Waters Corporation, Milford, MA, USA) column of the following dimensions: 2.1 × 100 mm, 1.7 μm. The mobile phase was composed of methanol (A) and water (0.1% formic acid) (B). A gradient program was used as follows: 0–1 min, 10% A; 1–3 min, 10–35% A; 3–6 min, 35–65% A; 6–9 min, 65–85% A; 9–10 min, 85–90% A; 10–11 min, 90–10% A; 11–13 min, 10% A. The flow rate was 0.3 mL·min**^−^**^1^ and the injection volume was 1 μL. The column temperature was 35 °C. The chromatography reagents of the mobile phase were purchased from Fisher Scientific, Loughborough, UK.

#### 2.6.2. Q-TOF-MS Analysis

A Xevo G2-S Q Tof time-of-flight mass spectrometer (Waters Corporation, Milford, MA, USA) was used with an electrospray ionization (ESI) system. The ion source temperature was set at 120 °C and the desolvation gas temperature was adjusted to 400 °C. The desolvation gas flow was set at 1000 L h^−1^. The capillary voltage was 2.5 kV for the positive ion mode and 2.5 kV for the negative ion mode; the cone voltage was set at 25 V, whereas the detection of the *m*/*z* ratio between 100 and 1200 was adjusted to positive and negative ionization.

#### 2.6.3. Data Collection and Analysis

The software Masslynx 4.1 was used to process the chromatograms in the negative ESI mode. The elemental composition was used to obtain both molecular and fragment ions. The UNIFI^®^ Scientific Information System (Waters Corporation, Milford, MA, USA) was used to analyze the structure of the chemical compositions.

### 2.7. Statistical Analysis

All data were analyzed using SPSS 13.0 (SPSS, Inc., Chicago, IL, USA) by one-way analysis of variance. The post hoc test was used for the least significance differences test. The significance level of the differences was set at *p* < 0.05.

## 3. Results

### 3.1. Estimation of Harmful Algae Density of P. globosa Treated by Mangrove Leaf Aqueous Extracts of Different Leaf Ages

**Figure 2:**
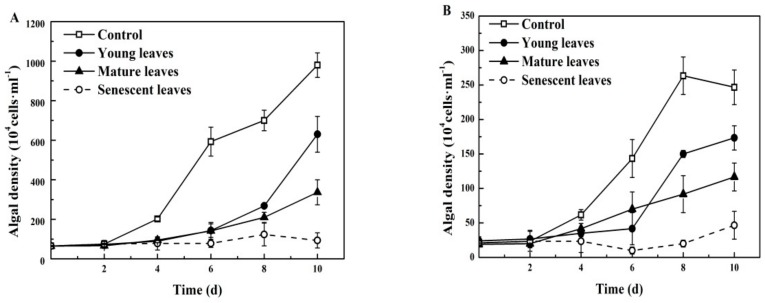
Harmful algae density of *P. globosa* following treatment with mangrove leaf aqueous extracts of different leaf age: (**A**) *B. gymnorrhiza* and (**B**) *K. candel*. The data are expressed as the mean ± standard error of the mean; *n* = 3 in each group.

The inhibitory effects of the dry powder aqueous extracts of the leaves of *B. gymnorrhiza* at different leaf age on *P. globosa* were significantly different following four days of exposure (Figure 2A). During days 4 to 8 of treatment, the differences between the four groups were gradually evident, and the algal cell density of the three treatment groups was significantly lower than of the control group (*p* < 0.05). On day 10, the inhibitory effects on *P. globosa* were in the following order: senescent (93.33 × 10^4^ cells·mL**^−^**^1^) > mature (336.67 × 10^4^ cells·mL**^−^**^1^) > young leaves (630.00 × 10^4^ cells·mL**^−^**^1^).

On day four, the inhibitory effects of the *K. candel* leaves with different leaf age on *P. globosa* were significant (*p* < 0.05), and the differences in the effects between the four groups were gradually evident (Figure 2B). The algal cell densities of the three treatment groups were significantly lower than those of the control group (*p* < 0.05). On day six, a significant difference in algal cell density between mature and senescent leaf groups was observed (*p* < 0.05). On day 8, significant differences occurred between the four groups (*p* < 0.05) as follows: young leaves (173.33 × 10^4^ cells·mL**^−^**^1^) > mature (116.67 × 10^4^ cells·mL**^−^**^1^) > senescent (46.67 × 10^4^ cells·mL**^−^**^1^).

### 3.2. Physiological Measurements

**Figure 3:**
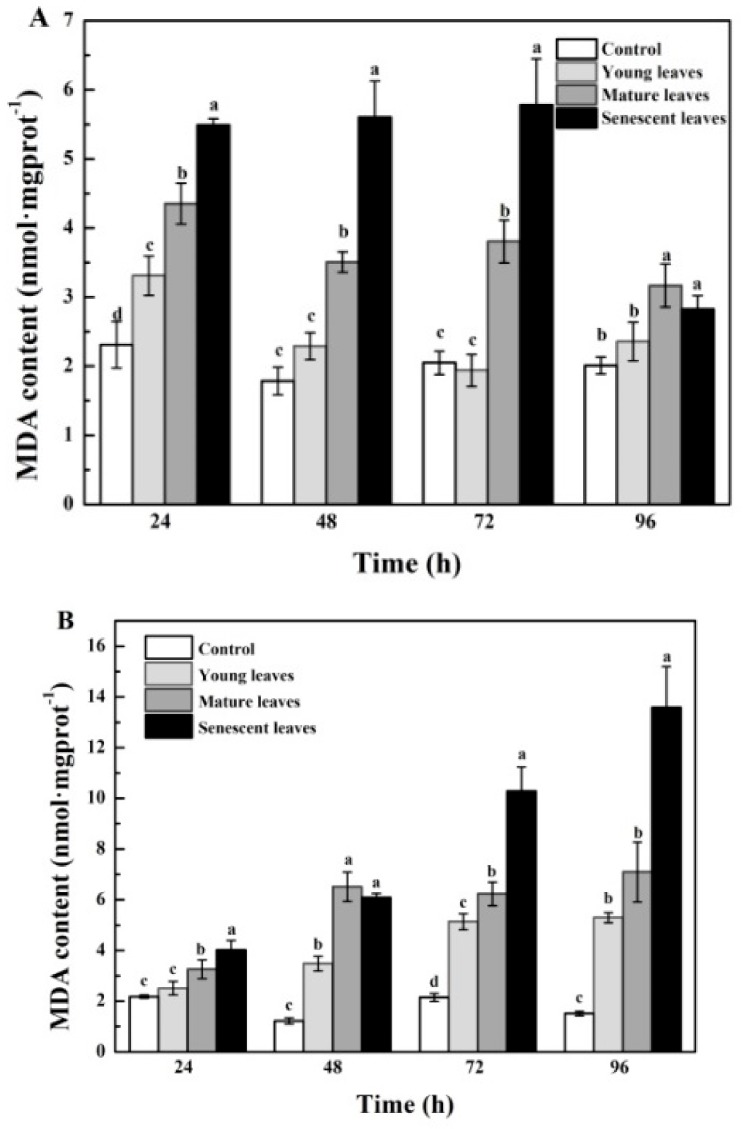
Malondialdehyde (MDA) content in *P. globosa* following treatment with mangrove leaf aqueous extracts of different leaf ages: (**A**) *B. gymnorrhiza* and (**B**) *K. candel*. The data are expressed as the mean ± standard error of the mean; *n* = 3 in each group. Values with different letters differ significantly from each other in the same time (*p* < 0.05).

The MDA content of *P. globosa* showed an upward trend with the aging of the mangrove leaves for the *B. gymnorrhiza* treatment groups (Figure 3A). The highest MDA content of *P. globosa* was noted for the senescent leaf group, followed by the mature and young leaf groups. Figure 3B demonstrated the effects of *K. candel* leaf extracts on the MDA content in *P. globosa*. The MDA content of *P. globosa* increased in the three *K. candel* treatment groups. The MDA content of *P. globosa* in the senescent leaf group was higher than that of the other groups (*p* < 0.05). The data indicated that the MDA contents in the three treatment groups were in the following order: senescent > mature > young leaves.

**Figure 4:**
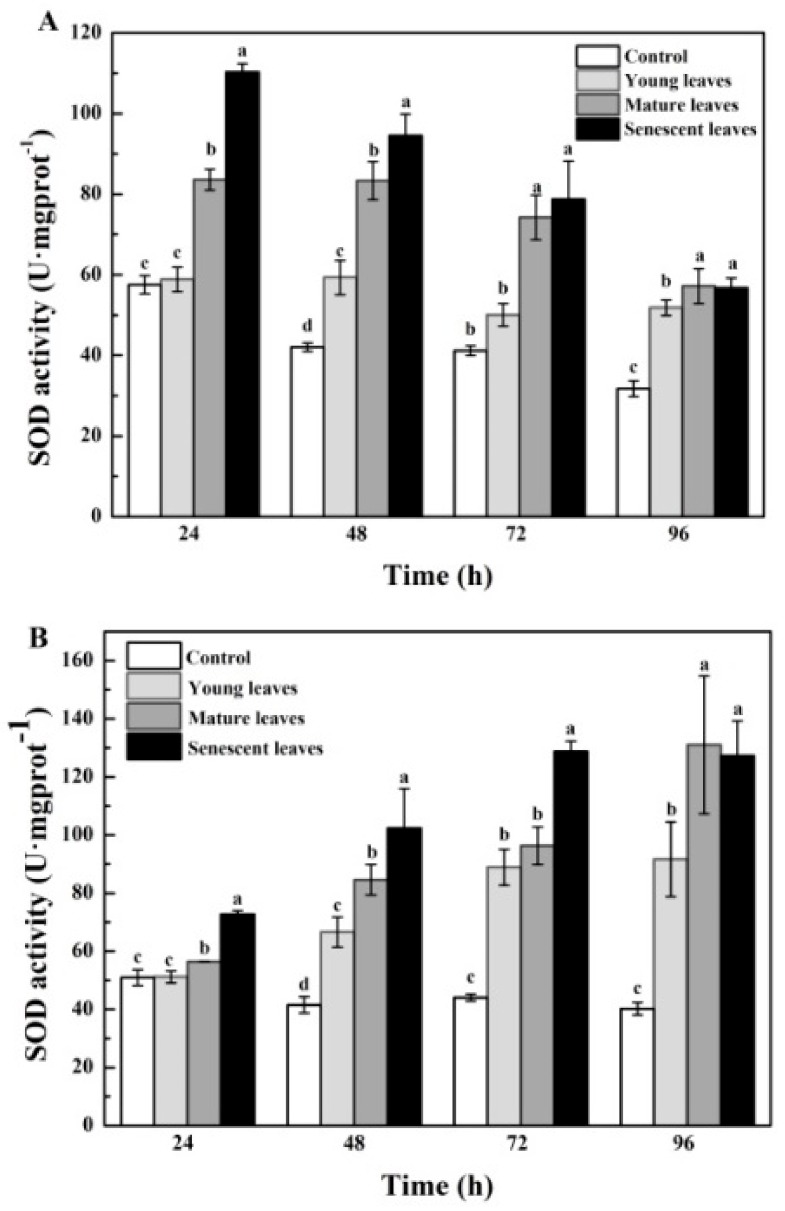
Superoxide dismutase (SOD) activity in *P. globosa* following treatment with mangrove leaf aqueous extracts of different leaf ages: (**A**) *B. gymnorrhiza* and (**B**) *K. candel*. The data are expressed as the mean ± standard error of the mean; *n* = 3 in each group. Values with different letters differ significantly from each other in the same time (*p* < 0.05).

Figure 4A indicated the effects of *B. gymnorrhiza* leaf extracts on the SOD activities in *P. globosa*. SOD activities decreased in senescent and mature leaves groups following an increase in the treatment time. The SOD activities of *P. globosa* in the three treatment groups were significantly different than those of the control group and exhibited the following order: senescent > mature > young leaves. Significant differences were noted (*p* < 0.05). Figure 4B indicated the effects of *K. candel* leaf extracts on the SOD activities of *P. globosa*. From the beginning to the end of the tests, the SOD activities of *P. globosa* were increased in the treatment groups. The data suggested that the SOD activities of *P. globosa* in the three treatment groups showed significant differences compared with those of the control group and were ranked as follows: senescent > mature > young leaves (*p* < 0.05).

**Figure 5:**
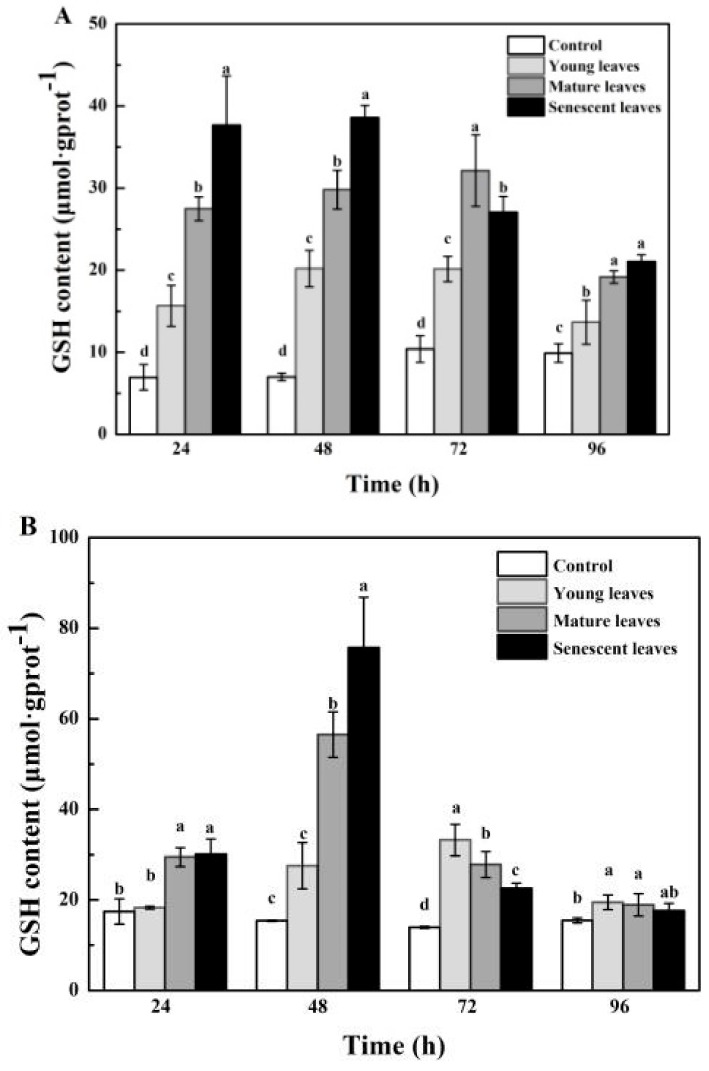
Glutathione (GSH) content in *P. globosa* following treatment with mangrove leaf aqueous extracts of different leaf ages: (**A**) *B. gymnorrhiza* and (**B**) *K. candel*. The data are expressed as the mean ± standard error of the mean; *n* = 3 in each group. Values with different letters differ significantly from each other in the same time (*p* < 0.05).

The effects of *B. gymnorrhiza* leaf extracts at different growth stages on the GSH content in *P. globosa* are shown in Figure 5A. At 48 h, the content of senescent leaf groups reached a maximum of 38.58 µmol·g^−1^ prot, and the GSH content of *P. globosa* in the three treatment groups showed significant differences (*p* < 0.05). Figure 5B showed the effects of *K. candel* leaf extracts on GSH content in *P. globosa*. The GSH content of the experimental groups of *K. candel* leaf extracts showed an upward trend with time and was subsequently decreased. The content of both senescent and mature leaf groups reached a maximum at 48 h (56.53 and 75.68 µmol·g^−1^ prot, respectively), and the GSH content of *P. globosa* in the three treatment groups indicated significant differences compared with that of the control samples (*p* < 0.05).

### 3.3. Identification of Chemical Components

**Figure 6:**
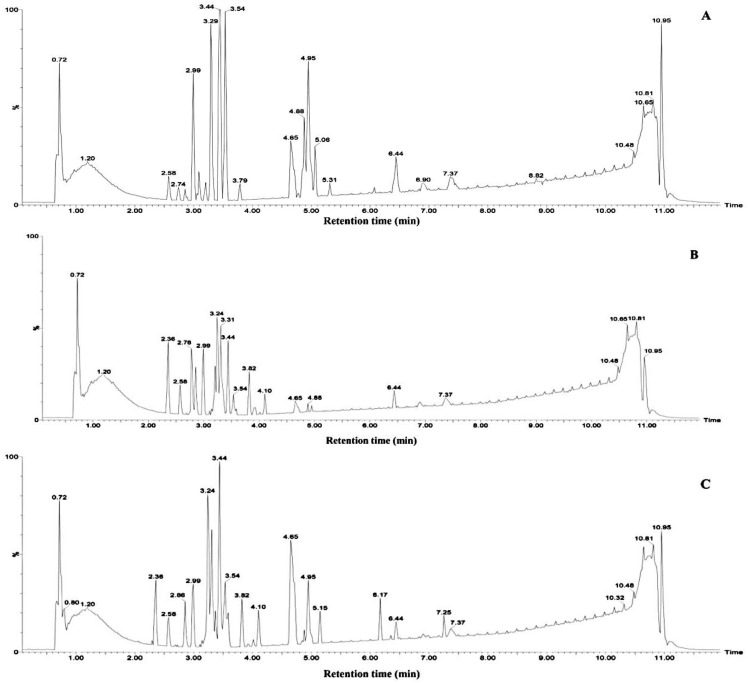
Identification of chemical constituents from *B. gymnorrhiza* leaf aqueous extracts in negative mode. (**A**) Young leaf, (**B**) Mature leaf, (**C**) Senescent leaf. Numbers above peaks represent retention times, in minutes.

**Figure 7:**
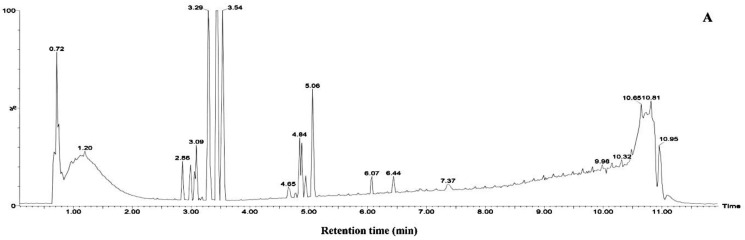

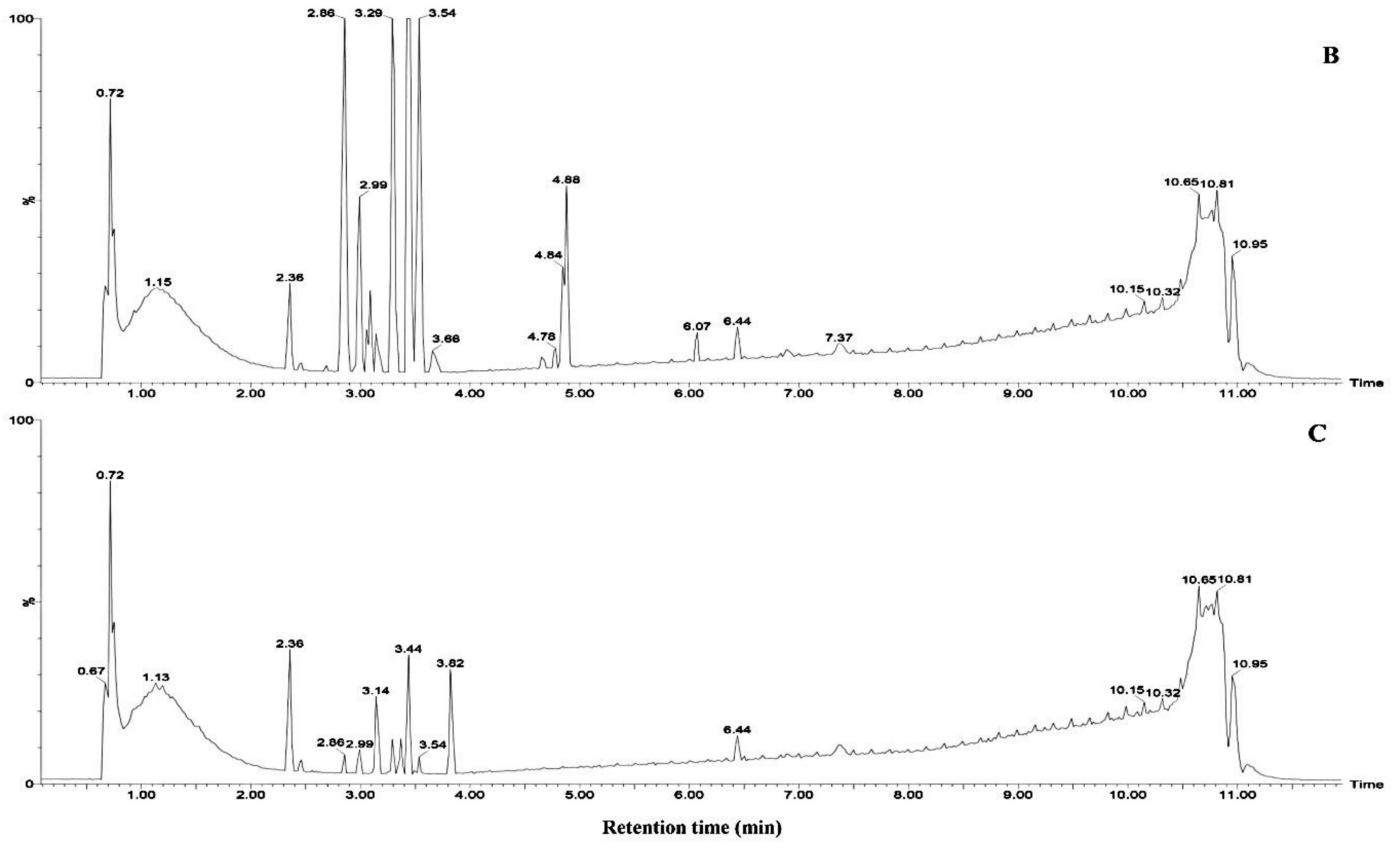
Identification of chemical constituents from *K. candel* leaf aqueous extracts in negative mode. (**A**) Young leaf, (**B**) Mature leaf, (**C**) Senescent leaf. Numbers above peaks represent retention times, in minutes.

**Figure 8:**
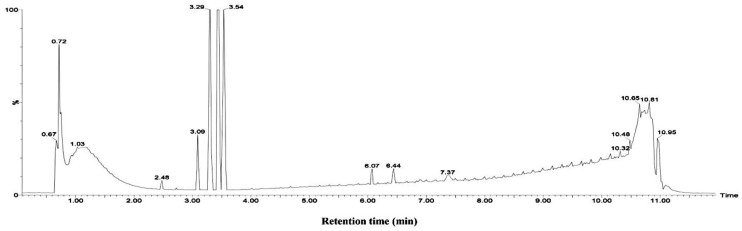
Identification of chemical constituents from sterilized artificial seawater in negative mode. Numbers above peaks represent retention times, in minutes.

**Figure 9:**
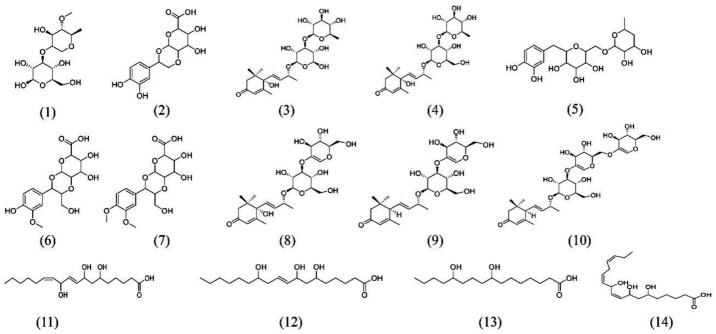
Chemical structures of compounds **1**–**14**.

The results derived from the negative ion mode were considerably better than those from the positive ion mode. Therefore, the present study used negative ion mode to analyze the chemical constituents of the aqueous extracts from the mangrove leaves. Excluded the chemical constituents found in the sterilized artificial seawater, a total of 14 types of major chemical constituents were detected in the leaf aqueous extracts of *B. gymnorrhiza* (Figure 6) and were identified as follows: 5 norsesquiterpenes compounds **3**, **4**, **8**, **9** and **10**; 4 phenolic glycoside compounds **2**, **5**, **6** and **7**; 1 carbohydrate compound **1**; And 4 fatty acid compounds **11**, **12**, **13** and **14**. A total of 11 types of major chemical constituents were detected in the leaf aqueous extracts of *K. candel* (Figure 7) and were identified as follows: 3 norsesquiterpenes compounds **4**, **8** and **9**; 3 phenolic glycoside compounds **5**, **6** and **7**; 1 carbohydrate compound **1** and **4** fatty acid compounds **11**, **12**, **13** and **14**. The name of the chemical constituents were in Table 1.The identified chemical constituents, namely the norsesquiterpenes compounds and the phenolic glycosides are new structures that have not been reported before. All the chemical constituents of the 5 norsesquiterpenes compounds were analogs of the compound roseoside **II**. The aglycon of the compound roseoside **II** was vomifoliol, following identification of the aqueous extracts of *B. gymnorrhiza* and *K. candel* leaves that contained vomifoliol. Due to the small amount of vomifoliol in the aqueous extracts, only a small chromatographic peak could be observed in the chromatogram (Rt 5.67). Figure 8 showed the chemical constituents found in the sterilized artificial seawater and Figure 9 showed the chemical structures of compounds **1**–**14**.

Using the UNIFI^®^ Scientific Information System, the relative content comparison of the chemical components of different experimental groups was carried out by the intensity of the ion peaks. Table 1 indicated that with the increase of the leaf age, the intensities of the ion peaks corresponding to the four types of norsesquiterpenes compounds **4**, **8**, **9** and **10** and to the phenolic glycoside compound **7** in the *B. gymnorrhiza* leaf aqueous extracts were gradually increased. The concentration levels of one norsesquiterpenes compound that was assigned **8**, one phenol glycoside compound assigned **7** and one carbohydrate compound assigned **1** in the *K. candel* leaves aqueous extracts were gradually increased.

## 4. Discussion

### 4.1. Inhibition of Aqueous Extract on Harmful Algae

In the present study, the two species of mangrove leaf aqueous extracts demonstrated significant inhibition on the red tide algae *P. globosa*, which was similar to the findings reported by previous studies. Sun et al. [21] reported that *B. gymnorrhiza* leaf extracts exhibited significant inhibition of *P. globosa* growth, and Chen and Peng [42] demonstrated that three mangrove species (*Avicennia marina*, *Aegicerax corniculata*, and *B. gymnorrhiza*) exhibited growth inhibition of radish (*Raphanus sativus*) and lettuce (*Lactuca sativa)* algae. We observed that the dry-powder aqueous extracts of senescent, mature, and young mangrove leaves had different inhibitory effects on the growth of *P. globosa*. As leaves became senescent, an increasing inhibitory effect was noted on the leaf extracts with regard to the growth of *P. globosa*. Thus, it is likely that the content of several substances in the mangrove leaves increased during their aging. Makkar et al. [43] highlighted that oak leaves had increased levels of certain substances according to their maturity, which was similar to the results obtained in this experiment.

### 4.2. Inhibition of Aqueous Extract on Antioxidant Defense System in Harmful Algae

In the present study, the aging of the leaves increased the MDA contents in *P. globosa*. The upward trend of the MDA contents indicated that the active oxygen and its products exhibited a potent oxidizing ability and a destructive effect on several biomolecules [31]. Reactive oxygen can convert fatty acids into toxic peroxides [44], destroy biofilms [45], and cause accumulation of MDA [46]. Following prolonged exposure, the effect of *K. candel* leaf extracts on MDA content in *P. globosa* gradually increased, indicating that the degree of lipid peroxidation of algal cells was continuously enhanced, and that the excessive accumulation of active oxygen in *P. globosa* may have caused oxidative stress [47]. Following prolonged exposure, the MDA content in *P. globosa* treated with *B. gymnorrhiza* leaf extracts showed a downward trend, indicating that the antioxidant defense system in the cells had gradually removed MDA levels [48]. Similar findings have been previously shown with regard to low temperature stress on the antioxidant system and the photosynthetic apparatus of *Kappaphycus alvarezii*, which is in agreement with the results presented in the current study [49].

Moreover, the data indicated that the aging of the leaves decreased the SOD activities in *P. globosa* as demonstrated by the effect of the extracts from the leaves of *K. candel* and *B. gymnorrhiza.* The upward trend of the SOD activities indicated that algal cells exhibited an antioxidant defense system and that the activity of their protective enzymatic system could be enhanced to remove harmful products [50]. Following prolonged exposure time, the effect of *K. candel* leaf extracts on SOD in *P. globosa* gradually increased, indicating that the degree of lipid peroxidation of algal cells was continuously enhanced [51]. This resulted in excessive accumulation of active oxygen in *P. globosa* and consequently increased oxidative stress [52]. Consequently, SOD activity in *P. globosa* increased in order to reduce the damage caused by active oxygen. Previous studies indicated similar results [53,54]. Under the effect of *K. candel* leaf extracts at 96 h, SOD activity in *P. globosa* was slightly decreased and was lower than that of the mature leaf group. This occurred due to the potent damage caused to the antioxidant enzyme system by the *K. candel* senescent leaf extracts and by the cell membranes of *P. globosa*, which led to a decrease in SOD activity [55].

The GSH content is related to the activity of the metabolic enzymes [34]. Prolonged exposure resulted in gradually reduced effects of mangrove leaf extracts on GSH in *P. globosa*, indicating that when the aqueous extract of the leaves of mangrove plants acted on algae cells, these cells could induce their non-enzymatic antioxidant system to counteract the damage caused by the aqueous extract [56]. Non-enzymatic antioxidants, such as GSH, directly capture free radicals in *P. globosa* to resist oxidative stress [48]. In the *K. candel* treatment groups, 24 h of cell exposure to the inhibitory substances of the extracts resulted in production of large amounts of free radicals in the algal cells. Subsequently, the GSH consumption was accelerated due to the increased oxidative stress caused from free radicals, and thus GSH content declined. Our results were similar to those of a previous study on bacterial substances and growth inhibition of *P. globosa* [54].

### 4.3. Analysis of Chemical Components

The results of the current analysis indicated that the chemical constituents of the aqueous extracts of the *B. gymnorrhiza* leaves were similar to those of the aqueous extracts of the *K. candel* leaves, and that these constituents were mainly high polarity glycoside-based compounds. These findings are different from other reports conducted on the chemical constituents of *B. gymnorrhiza* and *K. candel* [57]. This is because, in the present study, the chemical components of the leaves were all originated from mangrove plants and extracted with water [58]. However, the majority of the components reported in the previous studies were of low polarity or semipolar substances extracted with petroleum ether or ethyl acetate following initial extraction by methanol and/or ethanol [59,60].

The current study demonstrated that the levels of specific compounds were increased with the increase of leaf age. Notably, the following compounds showed elevated concentrations: Four types of norsesquiterpenes compounds assigned **4**, **8**, **9** and **10**, a phenolic glycoside compound assigned **7** in the *B. gymnorrhiza* leaf aqueous extracts, one norsesquiterpenes compound assigned **8**, one phenol glycoside compound assigned **7** and one carbohydrate compound assigned **1** in the *K. candel* leaf aqueous extracts. As leaves became senescent, an increasing inhibitory effect of leaf extracts was noted on the growth of *P. globosa*, and so it was likely that the content of some substances which could inhibit the growth of *P. globosa* in mangrove leaves increased during their aging.

Secondary metabolites in the mangrove plants play an essential role in improving plant self-protection and survival competitiveness [61]. Due to various factors, with the aging of the mangrove plant leaves, the leaves gradually stop growing, and the photosynthesis and respiration of mangrove plant leaves gradually weaken [62]. Some secondary metabolites accumulate in the leaf tissue did not metastasize or degrade [63]. Therefore, some secondary metabolites obtained in the present study assigned **4**, **7**, **8**, **9** and **10** continue to accumulate in the mangrove leaves with the increase of the leaf age. In the present study, carbohydrate compound **1** was identified as primary metabolites, which were generally associated with the nutrition and development of plants [64]. However, the majority of the substances that inhibited the growth of the algae that were found in mangrove plants were secondary metabolites [65]. Therefore, we considered that the secondary metabolites obtained in the current study, namely the 4 types of the norsesquiterpenes compounds assigned **4**, **8**, **9** and **10** and the phenolic glycoside compound **7**, exerted the inhibitory effect on the growth of algae. Previous studies have confirmed that phenolic glycosides in *Rubus ulmifolius* could effectively inhibit the growth of *aureus* [66]. Macías et al. [67] reported that 6 types of secondary metabolites (Norsesquiterpenes compounds) extracted from sunflower can exhibit significant inhibitory effects on the growth of specific plants. These results validate the conclusions of the present study. In addition, it was speculated that the substance **13** in the aqueous extract of the leaves of *B. gymnorrhiza* and the components **5**, **11**, **12** and **13** in the aqueous extract of the leaves of *K. candel* had no associations with the inhibition of the growth of the red tide algae as their levels were continuously reduced with the increase of the leaf age.

## 5. Conclusions

The leaf extracts of two mangrove species, namely *Kandelia candel* and *Bruguiera gymnorrhiza* exhibited inhibitory effects on the growth of red tide algae *P. globosa*. In addition, the extracts from leaves with different age exhibited different inhibitory effects on *P. globosa,* and with the aging of the leaves, the inhibitory effects showed an increasing trend. Senescent leaves exhibited the highest inhibitory effects. We consider that the secondary metabolites that were identified in the present study (Compounds **4**, **8**, **9,** and **10** and a phenolic glycoside compound **7**) exerted the inhibitory effect on the growth of the algae.

## Figures and Tables

**Table 1:** Identification of chemical constituents from *B. gymnorrhiza* and *K. candel* leaves aqueous extracts.

No.	Rt (min)	Name of the Chemical Constituent	M/Z	Types	Molecular Formula	Ion Peak Intensity					
						*Bruguiera gymnorrhiza* Leaves			*Kandelia candel* Leaves		
						Young	Mature	Senescent	Young	Mature	Senescent
1	2.36	(2R,3R,4S,5R,6R)-4-(((4R,5S,6R)-4-hydroxy-5-methoxy-6-methyltetrahydro-2H-pyran-3-yl)oxy)-6-(hydroxymethyl)tetrahydro-2H-pyran-2,3,5-triol	323.1334	Carbohydrate compound	C_13_H_24_O_9_	--	149,287	129,132	23,013	105,564	127,743
2	2.58	3-(3,4-dihydroxyphenyl)-7,8-dihydroxyhexahydro-6H-pyrano[2,3-b][1,4]dioxine-6-carboxylic acid	327.0695	Phenolic glycoside compound	C_14_H_16_O_9_	92,476	116,958	113,029	--	--	--
3	2.78	(S)-4-hydroxy-3,5,5-trimethyl-4-((R,E)-3-(((2R,3R,4S,5S,6S)-3,5,6-trihydroxy-4-(((2S,3R,4S,5S,6R)-3,4,5-trihydroxy-6-methyltetrahydro-2H-pyran-2-yl)oxy)tetrahydro-2H-pyran-2-yl)oxy)but-1-en-1-yl)cyclohex-2-en-1-one	517.2288553.2073	Norsesquiterpenes compound	C_24_H_38_O_12_	--	245,060	15,034	--	--	--
4	2.83	(S)-4-((R,E)-3-(((2R,3R,4S,5R,6R)-3,5-dihydroxy-6-(hydroxymethyl)-4-(((2S,3R,4S,5S,6R)-3,4,5-trihydroxy-6-methyltetrahydro-2H-pyran-2-yl)oxy)tetrahydro-2H-pyran-2-yl)oxy)but-1-en-1-yl)-4-hydroxy-3,5,5-trimethylcyclohex-2-en-1-one	531.2444567.2008	Norsesquiterpenes compound	C_25_H_40_O_12_	--	25,139	31,151	--	2287	--
5	2.86	2-(((3,4-dihydroxy-6-methyltetrahydro-2H-pyran-2-yl)oxy)methyl)-6-(3,4-dihydroxybenzyl)tetrahydro-2H-pyran-3,4,5-triol	415.1601	Phenolic glycoside compound	C_19_H_28_O_10_	39,667	109,141	97,293	89,675	26,140	--
6	2.99	7,8-dihydroxy-3-(4-hydroxy-3-methoxyphenyl)-2-(hydroxymethyl)hexahydro-6H-pyrano[2,3-b][1,4]dioxine-6-carboxylic acid	371.0981	Phenolic glycoside compound	C_16_H_20_O_10_	97,120	92,547	143,956	89,789	195,885	48,488
7	3.14	3-(3,4-dimethoxyphenyl)-7,8-dihydroxy-2-(hydroxymethyl)hexahydro-6H-pyrano[2,3-b][1,4]dioxine-6-carboxylic acid	386.1213	Phenolic glycoside compound	C_17_H_22_O_10_	32,390	55,871	57,402	49,357	92,773	120,291
8	3.24	(S)-4-((R,E)-3-(((2R,3R,4S,5R,6R)-4-(((2R,3S,4S)-3,4-dihydroxy-2-(hydroxymethyl)-3,4-dihydro-2H-pyran-5-yl)oxy)-3,5-dihydroxy-6-(hydroxymethyl)tetrahydro-2H-pyran-2-yl)oxy)but-1-en-1-yl)-4-hydroxy-3,5,5-trimethylcyclohex-2-en-1-one	529.2261	Norsesquiterpenes compound	C_25_H_38_O_12_	9285	366,457	488,103	--	10,581	11,959
9	3.82	(R)-4-((R,E)-3-(((2R,3R,4S,5R,6R)-4-(((2R,3S,4S)-3,4-dihydroxy-2-(hydroxymethyl)-3,4-dihydro-2H-pyran-5-yl)oxy)-3,5-dihydroxy-6-(hydroxymethyl)tetrahydro-2H-pyran-2-yl)oxy)but-1-en-1-yl)-3,5,5-trimethylcyclohex-2-en-1-one	513.2323	Norsesquiterpenes compound	C_25_H_38_O_11_	11,577	127,005	153,792	--	--	2576
10	4.10	(R)-4-((R,E)-3-(((2R,3R,4S,5R,6R)-4-(((2R,3S,4S)-2-((((2R,3S,4S)-3,4-dihydroxy-2-(hydroxymethyl)-3,4-dihydro-2H-pyran-5-yl)oxy)methyl)-3,4-dihydroxy-3,4-dihydro-2H-pyran-5-yl)oxy)-3,5-dihydroxy-6-(hydroxymethyl)tetrahydro-2H-pyran-2-yl)oxy)but-1-en-1-yl)-3,5,5-trimethylcyclohex-2-en-1-one	657.2775	Norsesquiterpenes compound	C_31_H_46_O_15_	19,794	126,772	183,038	--	--	--
11	4.65	(9E,12Z)-6,8,11-trihydroxyoctadeca-9,12-dienoic acid	327.2176	Trihydroxy linoleic acid(fatty acid compound)	C_18_H_32_O_5_	257,947	114,223	439,706	91,241	71,045	3746
12	4.95	(E)-6,8,12-trihydroxyoctadec-9-enoic acid	329.2322	Trihydroxy oleic acid(fatty acid compound)	C_18_H_34_O_5_	404,377	64,238	212,648	108,359	39,937	--
13	5.06	8,12-dihydroxyhexadecanoic acid	287.2212	Dihydroxy palmitic acid(fatty acid compound)	C_16_H_32_O_4_	125,551	7562	6723	221,948	5952	--
14	5.15	(9Z,12Z,15Z)-6,8,11-trihydroxyoctadeca-9,12,15-trienoic acid	325.2018	Trihydroxy linolenic acid(fatty acid compound)	C_18_H_30_O_5_	26,099	19,226	99,204	6470	--	-
